# The effect of a weight gain prevention intervention on moderate-vigorous physical activity among black women: the Shape Program

**DOI:** 10.1186/s12966-017-0596-6

**Published:** 2017-10-16

**Authors:** Mary L. Greaney, Sandy Askew, Sherrie F. Wallington, Perry B. Foley, Lisa M. Quintiliani, Gary G. Bennett

**Affiliations:** 1 Department of Kinesiology and Health Studies, 25 West Independence Way, University of Rhode Island, Kingston, RI 02881 USA; 2 Duke Global Digital Health Science Center, Duke Global Health Institute, Duke University, Durham, NC 27710 USA; 3 Georgetown Lombardi Comprehensive Cancer Center, Georgetown University, Washington, D.C., 20007 USA; 4 Section of General Internal Medicine, Department of Medicine, Boston University, Boston, MA 02118 USA; 5 Department of Psychology and Neuroscience, Duke University, Durham, NC 27708 USA

**Keywords:** Accelerometers, Black women, Exercise, Accelerometers, Obesity

## Abstract

**Background:**

Rates of physical inactivity are high among Black women living in the United States with overweight or obesity, especially those living in the rural South. This study was conducted to determine if an efficacious weight gain prevention intervention increased moderate-vigorous physical activity (MVPA).

**Methods:**

The Shape Program, a weight gain prevention intervention implemented in community health centers in rural North Carolina, was designed for socioeconomically disadvantaged Black women with overweight or obesity. MVPA was measured using accelerometers, and summarized into 1- and 10-min bouts. We employed analyses of covariance (ANCOVA) to assess the relationship between changes in MVPA over 12 months, calculated as a change score, and intervention assignment (intervention versus usual care).

**Results:**

Participants completing both baseline and 12-month accelerometer assessments (*n* = 121) had a mean age of 36.1 (SD = 5.43) years and a mean body mass index of 30.24 kg/m^2^ (SD = 2.60). At baseline, 38% met the physical activity recommendation (150 min of MVPA/week) when assessed using 10-min bouts, and 76% met the recommendation when assessed using 1-min bouts. There were no significant differences in change in MVPA participation among participants randomized to the intervention from baseline to 12-months using 1-min bouts (adjusted intervention mean [95% CI]: 20.50 [−109.09 to 150.10] vs. adjusted usual care mean [95% CI]: -80.04 [−209.21 to 49.13], *P* = .29), or 10-min bouts (adjusted intervention mean [95% CI]: 7.39 [−83.57 to 98.35] vs. adjusted usual care mean [95% CI]: -17.26 [−107.93 to 73.40], *P* = .70).

**Conclusions:**

Although prior research determined that the Shape intervention promoted weight gain prevention, MVPA did not increase significantly among intervention participants from baseline to 12 months. The classification of bouts had a marked effect on the prevalence estimates of those meeting physical activity recommendations. More research is needed to understand how to promote increased MVPA in weight gain prevention interventions.

**Trial registration:**

This study is registered at www.clinicaltrials.gov database (No. NCT00938535. Retrospectively Registered 7/10/2009).

## Background

Black women in the United States are disproportionately affected by the obesity epidemic [1–3] with four out of every five Black women having overweight or obesity [2]. Obesity rates are greater among Black adults living in rural areas of the United States than in urban areas (56% versus 43%) [4]. Black women have low levels of physical activity and high rates of sedentary behavior compared to women in other racial/ethnic groups [5, 6], which increases their risk of negative health outcomes associated with obesity, physical inactivity, and sedentary behaviors [7–11].

Adults often gain an average of 0.5–1 kg per year throughout middle age [12, 13], with Black women gaining weight at a greater rate than White women. Weight loss approaches often have only modest impacts; only about 20% of people sustain their weight loss [14]. Therefore, a new paradigm may be useful [15]. Weight gain prevention interventions that focus on weight maintenance and not weight loss may be a valuable new approach to ameliorate or reduce obesity. This approach may be particularly salient for Black women with overweight or obesity, since they have a greater tolerance of a heavier body size [16].

Participation in moderate-vigorous physical activity (MVPA) may be an important strategy for preventing weight gain [17–20]. However, only a limited number of weight gain prevention interventions have focused on and/or have assessed the impact of the intervention on participants’ physical activity [21]. Research in this area is needed because physical activity is a weight maintenance strategy that may have important spillover health benefits. In order to address this gap, we examined the impact of The Shape Program, a weight gain prevention program designed for Black women living in the rural South with overweight or obesity [15, 22], on MVPA.

## Methods

### The Shape Program

The Shape Program (henceforth referred to as Shape) was a randomized controlled trial of a 12-month weight gain prevention intervention implemented in rural North Carolina community health centers between 2009 and 2012. The intervention prevented weight gain at 12 and 18 months [22] and has been described in detail elsewhere [15]. Participants were Black female patients aged 25–44 with a body mass index (BMI) of 25–34.9 kg/m^2^ who had made at least one visit to the participating health care system in the previous 24 months, and were fluent in English.

### Ethics, consent, and permission

Participants provided signed informed consents and completed baseline assessments. Next, they were fitted with accelerometers, and then randomized to either the intervention arm or usual care. Participants completed additional assessments at 12 months post-enrollment and were again fitted with accelerometers. The study was approved by the Institutional Review Board at Duke University.

### The Shape intervention

The Shape intervention was developed using social cognitive theory [23], with self-efficacy selected as the primary mediator [15]. The intervention utilized the interactive obesity treatment approach (iOTA), where individuals self-monitor concise and easy to comprehend tailored behavior change goals. iOTA reduces literacy and numeracy issues that may occur in traditional lifestyle interventions for weight management [15, 22]. Individuals randomized to the intervention arm received: (a) tailored behavior change goals to promote the prevention of weight gain; (b) skills training materials; (c) weekly interactive voice response (IVR) telephone calls for self-monitoring behaviors; (d) monthly telephone coaching from a registered dietitian trained in motivational interviewing; and (e) a no-cost 12-month membership to a YMCA facility of their choice. Based on our prior work [24, 25], participants were assigned goals (versus selecting their own) to maximize the likelihood of success. Goals were tailored to the individual using an algorithm accounting for the participant’s need for change, self-efficacy, and readiness for change. Goals were updated every 8 weeks based on output from the original algorithm, with one goal always focused on physical activity. For example, one goal was to walk 7000 steps per day, which increased to 8000 and then 10,000 steps when the goal was regularly met. For the first 8 weeks, individuals were assigned three goals, and for the rest of the intervention period individuals were assigned four goals for each 8-week interval.

Printed skills training materials, tailored to participants’ assigned behavior change goals, were designed for low-literacy audiences [26] and included information (e.g., overcoming barriers to physical activity, portion sizes, food shopping tips, healthy recipes) and tracking logs specific to each assigned goals. The training materials also included information useful for behavior change regardless of the behavior (e.g., implementing social environmental change, managing time). Participants received their first set of skills training materials at the baseline assessment and were mailed additional materials every 8 weeks throughout the intervention period when new goals were assigned.

During the coaching calls, health coaches utilized principles of motivational interviewing when reviewing self-monitoring data and when assisting participants in their change efforts. They also offered participants information on a range of topics such as negotiating barriers, engaging social support, identifying ways to be physically active throughout the day, and maintaining motivation.

Although the intervention was implemented in the primary care setting, with the exception of a 12-month YMCA membership provided at no cost, intervention components were designed to be completed at participants’ homes. Participants randomized to usual care arm were mailed semi-annual newsletters during the intervention period. These newsletters covered general wellness topics but did not address physical activity, nutrition or weight.

### Measures

#### Physical activity

At the baseline and 12-month assessments, participants were fitted with accelerometers (Actical, Philips Respironics, Inc., Bend, OR, USA). Accelerometers were placed on participants’ non-dominant wrists using plastic locking wrist straps (like a wristwatch) that could only be removed by being cut off. Participants were asked to wear the accelerometers continuously until their return visit approximately 14 days later and were informed that the device could be worn while showering and sleeping and only needed to be removed if swimming for more than 30 min. The accelerometer data were screened and processed using procedures consistent with recent recommendations [27, 28]. Complete files were defined as those in which participants wore the monitor for seven or more days for at least 10 h of valid wear time per day. The raw data files were converted into minute-by-minute values of activity energy expenditure (AEE; kcal/kg/min) using a previously published calibration algorithm [29], and summarized into 1- and 10-min bouts of MVPA. We calculated 10-min bouts to align with current physical activity recommendations [30] and, given the low rates of Black women meeting physical activity recommendations [5, 6], we wanted to explore whether participants increased their MVPA if shorter bouts of MVPA were taken into account. Both 1-min and 10-min bouts were used to determine whether participants met current physical activity recommendations (150+ minutes of MVPA/week).

#### Health-related measures

Trained study staff measured weight to the nearest 0.1 kg using a portable electronic scale (Seca Model 876) and measured height using a calibrated wall-mounted stadiometer (Seca 214). Height and weight were then used to calculate body mass index (BMI). As part of the baseline survey, participants reported their perceived health status, which was assessed using the Medical Outcomes Study Short-Form; response categories were dichotomized (excellent/very good/good versus fair/poor) [31].

#### Demographic measures

Examined demographic variables included age, marital/partner status, educational attainment, employment status, income, and number of children in the household. The last two measures were used to determine if participants’ household incomes were above or below/borderline the federal poverty line based on the 2010 federal poverty guidelines for income and household size. For example, a family of three with a reported household income falling in the category of $10,000–$19,999 would be classified as being borderline/at the federal poverty level of $18,301 [32], while those with lower incomes were classified as being below the federal poverty level, and those with higher incomes were classified as being above the federal poverty level.

### Analysis

**Fig. 1:**
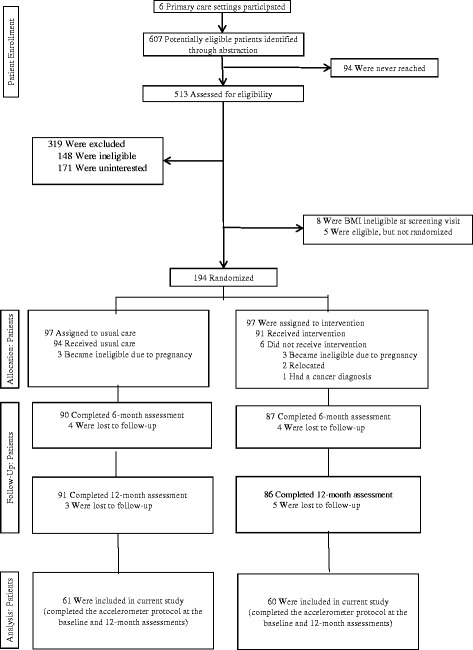
Consort Diagram

The analytic sample included participants who completed the accelerometer protocol at the baseline and 12-month assessments (*n* = 121, 63.3% of the baseline sample, see Fig. 1.). We calculated descriptive statistics for all key variables at baseline and used t-tests and chi square tests to determine if there were differences between the intervention and usual care groups in sociodemographics, health-related measures, MVPA participation, and in meeting the physical activity recommendation at baseline as assessed by 1-min and 10-min bouts. Likewise, we tested for differences between participants in our analysis sample and those participants excluded due to insufficient accelerometer data.

We employed analyses of covariance (ANCOVA) to assess the relationship between change in MVPA over 12 months, calculated as a change score, and intervention assignment (intervention versus usual care). We used step-wise model selection methods using *F*-tests (*P* = .20 entrance criteria and a *P* = .10 staying criteria) and several criterion-based procedures (best Akaike Information Criterion (AIC), Mallows C_p_, SBC or adjusted R^2^) to identify the best model from the health-related and demographic variable pool for change in total MVPA minutes per week using 10-min bouts. The pool consisted of available health-related measures (health status, BMI) and demographic characteristics (age, education, employment status, poverty level, marital/partner status and number of children in household) believed to potentially impact physical activity. Although the models identified by each technique were similar, slight variations were present; we selected the model created by our *F*-test sequence, which also had the best AIC and best Mallow’s C_p_ as the final model. This model included poverty level and BMI as covariates. We used a similar procedure for the 1-min bouts data, but none of these criteria agreed on a single best model. Therefore we selected the model suggested by our significance-based criteria, since it included poverty level and BMI as covariates, which was consistent with the 10-min bout model.

## Results

Participants (*n* = 121) had a mean age of 36.1 (SD = 5.43) years and a mean BMI of 30.24 kg/m^2^ (SD = 2.60) (See Table 1.). The majority was employed (78%), not married/partnered (70%), and had a family income that placed them borderline/at or below the federal poverty level (62%). Participants who were excluded due to missing data (*n* = 64) were younger (mean age ± SD: 34.1 ± 5.4 years versus 36.1 ± 5.4 years, *p* = .02) and less likely to be employed (59% vs. 78%, *p* = .01). There were also marginal differences in the likelihood of reporting health as good, very good or excellent (47% versus 62%, *p* = .054), and of having children (44% versus 29%, *p* = .051). No significant differences were observed in the remaining demographic or health-related measures between the analytic sample for this study and Shape participants who were excluded due to missing accelerometer data (data not shown). Furthermore, there were no significant differences in the demographic or health-related measures between the intervention group and usual care groups (see Table 1).

**Table 1:** Baseline characteristics of The Shape Program participants with baseline and 12-month accelerometer data (*n* = 121)

	Total (*n* = 121)		Usual Care (*n* = 61)		Intervention (*n* = 60)		
	*%*	*n*	*%*	*n*	*%*	*n*	*P* value
Demographics							
Age (mean years)^a^	36.12	5.43	35.62	5.76	36.62	5.07	.32
Education							.997
< high school (HS) diploma	10.08	12	10.00	6	10.17	6	
HS diploma or GED	28.57	34	28.33	17	28.81	17	
Some college or more	61.34	73	61.67	37	61.02	36	
Federal poverty level (FPL)							.90
Above FPL	37.29	44	35.59	21	38.89	23	
Borderline/at FPL	32.20	38	32.20	19	32.20	19	
Below FPL	30.51	36	32.20	19	28.81	17	
Marital/Partner status							.51
Currently married/partnered	29.91	35	32.76	19	27.12	16	
Not married/partnered	70.09	82	67.24	39	72.88	43	
Number of children in household							.20
None	70.54	79	64.81	35	75.86	44	
1 or more	29.46	33	35.19	19	24.14	14	
Employment status							.26
Employed (full or part time)	77.97	92	73.77	45	82.46	47	
Not employed	22.03	26	26.23	16	17.53	10	
Health-related variables							
BMI (kg/m^2^)^a^	30.24	2.60	30.27	2.37	30.22	2.76	.92
Perceived health status							.45
Good/Very Good/Excellent	61.67	74	58.55	35	65.00	39	
Fair/Poor	38.33	46	41.67	25	35.00	21	

*Note*:

^a^Means and standard deviations are presented (vs. % and n).^2^ = 150 min of moderate-vigorous PA/week

About one-third (38%) of participants met the physical activity recommendation at baseline when assessed using 10-min bouts; this percentage increased to 76% when assessed using 1-min bouts (see Table 2). Participants randomly assigned to the usual care group participated in more MVPA during the baseline measurement period (see Table 2). However, there were no significant differences in MVPA participation by either group at 12 months or in the percent of participants meeting the physical activity recommendation at either baseline or 12 months. Furthermore, there was no significant change in MVPA over the intervention period (see Table 3) or significant difference in changes in MVPA by group assignment over the intervention period (see Table 4).

**Table 2:** Moderate-vigorous physical activity (MVPA) participation of The Shape Program participants with baseline and 12-month accelerometer data (*n* = 121)

	Total (*n* = 121)		Usual Care (*n* = 61)		Intervention (*n* = 60)		*P* value
Met physical activity rec.	*%*	*n*	*%*	*n*	*%*	*n*	
Baseline (1-min bouts)	76.0	92	77.1	47	75.0	45	.83
Baseline (10-min bouts)	38.0	46	41.0	25	35.0	21	.58
12 months (1-min bouts)	69.4	84	75.4	46	63.3	38	.17
12-months (10-min bouts)	31.4	38	34.4	21	28.3	17	.56
Minutes MVPA/week	Mean	SD	Mean	SD	Mean	SD	
Baseline (1-min bouts)	438.69	445.0	546.11	549.90	329.47	266.88	.007
Baseline (10-min bouts)	202.65	308.05	262.33	387.18	141.98	182.00	.03
12-months (1-min bouts)	396.98	471.23	455.00	518.69	337.98	413.54	.17
12-months (10-min bouts)	189.17	327.43	237.54	396.62	140.00	230.65	.10

**Table 3:** Results from ANCOVA models estimating moderate-vigorous physical activity (MVPA) change over 12 months among participants in the Shape Program (*n* = 118)

	MVPA at 12 months (1-min bouts) final model				MVPA at 12 months (10-min bouts) final model			
	DF^a^	Type III SS	F value	*P* value	DF	Type III SS	F value	*P* value
Random assignment	1	297,540.98	1.19	.28	1	17,894.45	0.15	.07
BMI (kg/m^2^)	1	683,372.27	2.73	.10	1	284,526.03	2.31	.13
Federal poverty level	2	1,457,041.95	2.91	0.06	2	848,682.20	3.44	.04

*Note*:

^a^ *DF* Degrees of freedom

**Table 4:** Adjusted mean change in weekly moderate-vigorous physical activity (MVPA) among participants in the Shape Program by intervention status estimated by ANCOVA models (*n* = 118)

	Adjusted 12-month mean change in MVPA (95% CI), minutes/week			
	Intervention (*n* = 59)	Control (*n* = 59)	Between group difference	*P* value
1-min bouts	20.50 (−109.09, 150.10)	-80.04 (−209.21, 49.13)	100.54 (−82.20, 283.29)	.29
10-min bouts	7.39 (−83.57, 98.35)	-17.26 (−107.93, 73.40)	24.66 (−103.61, 152.92)	.70

We conducted a sensitivity analyses that excluded the 9.7% who participated in no moderate to vigorous activity at baseline to determine if the intervention resulted in increased MVPA among participants who were participating in some MVPA at baseline; results remained the same (data not shown).

## Discussion

Shape was a multicomponent intervention previously shown to prevent weight gain in a sample of socioeconomically disadvantaged Black women who have overweight or obesity [15], a population with low rates of physical activity [5]. Given the importance of physical activity for weight maintenance [17–20] and overall health benefits independent from weight [33], we wanted to determine whether the Shape intervention increased MVPA. Analyses determined that MVPA did not increase over the 12-month intervention period. This finding, combined with the known efficacy of the Shape intervention, suggests that the weight gain prevention may have been due to dietary changes rather than increases in MVPA. It is possible that participants viewed diet-related changes as being more valuable and/or easier to integrate into their daily lives. It also is conceivable that participants prioritized dietary change goals, since more of the Shape behavioral changes goals focused on dietary changes than on physical activity. Future research in the weight gain prevention intervention arena would be well poised to investigate these beliefs and attitudes. In addition, research is needed to understand how to stress the importance of physical activity for overall health and how to promote both MVPA and dietary changes in weight gain prevention interventions. For example, a recent review of interventions that compared the effects of presenting behaviors either simultaneously or sequentially found that both approaches appeared to be effective on one, but not both outcomes [34]. Thus, additional questions remain for the optimal design of multiple behavior interventions to encourage behavior change across all targeted outcomes.

This study adds to the small body of research examining whether physical activity changes during the course of a weight gain prevention intervention. A recent study of a 2-year weight gain prevention intervention for students attending community college found that the intervention did not increase physical activity or reduce sugar-sweetened beverage intake, but did promote reductions in fast food intake [35]. Another recent randomized controlled trial of a weight gain prevention intervention determined that more than half of the participants [62.0% of sample, 56.3% of completers] maintained their body weight over the 12-month intervention, but energy expenditure did not change [36]. Study participants (*n* = 87) were premenopausal women between the ages of 18–45 with a BMI >18.5 kg/m^2^; 12% were Black.

Interventions that have focused on increasing physical activity among predominantly Black participants have had limited success [37, 38]. The Shape intervention addressed possible structural barriers to physical activity due to lack of access and opportunities [39, 40] by providing intervention participants with a no-cost membership to a local YMCA with available child care vouchers. About three-quarters (70.3%, *n* = 64) of the women activated their YMCA memberships but use of the memberships was limited, with 42.2% (*n* = 27) of the women making no subsequent visits to the YMCA over the 12-month intervention period. Women living near/below the federal poverty line and those who met the physical activity guidelines (assessed using 10-min bouts) were more likely to visit the gym at least once after activating their memberships [41]. Although YMCA memberships were provided, the Shape intervention messages primarily emphasized lifestyle-based physical activity changes -- such as parking one’s car further away -- to gain additional steps. It is possible that Shape participants perceived themselves as already being physically active and that this message did not resonate.

Shape health coaches discussed with intervention participants the importance of physical activity and provided strategies such as planning ahead, time management, and seeking social support to promote physical activity. In retrospect, it may have been beneficial to place greater emphasis on accumulating MVPA in 10-min bouts because the classification of bouts had a marked effect on the prevalence estimates of those meeting physical activity recommendations; at baseline, 76% of participants were classified as meeting the recommendation when using 1-min bouts versus 38% when using 10-min bouts. Similarly, a validation study conducted by Wolin et al. [42] found notable differences in mean minutes of moderate physical activity per week among Black women living in public housing when assessed by 1-min or 10-min bouts [660 (SD = 345) versus 91 (SD = 110) minutes/week, respectively]. The increase in the percentage of participants classified as meeting the physical activity recommendation when 1-min bouts were used indicates that study participants did participate in MVPA, but there was a need for bouts of longer duration. Future intervention efforts should focus on helping individuals who participate in short (< 10-min) bouts of MVPA increase the duration of their MVPA.

Study findings should be considered in light of study strengths and limitations. Study strengths include the use of objectively measured MVPA as well as a sample that is often underrepresented in obesity prevention trials [43]. However, as the study was conducted with low-income Black women living in the South, results of the study may have limited generalizability to other overweight and obese populations. In addition, our sample was a subset of the entire study sample, and it is not known whether the inclusion of participants with insufficient accelerometer data may have had an impact on the study findings. It would be informative to conduct qualitative research to determine why participants did not complete the accelerometer protocol, as this research could provide insights into increasing compliance. Furthermore, despite being randomly assigned to study arms, individuals randomized to usual care participated in greater MVPA at baseline when assessed using 1-min and 10-min bouts, although there was no difference in meeting the physical activity recommendation by study arm. Lastly, this study focused on MVPA and did not investigate changes in light intensity physical activity.

## Conclusion

In summary, study results determined that Shape was not effective in increasing MVPA among a sample of Black women with overweight or obesity, although prior research determined that the Shape intervention prevented weight gain. Intervention participants may have focused on making dietary changes over increasing physical activity. Results of this study add to the limited extant research examining changes in physical activity occur during the course of a weight gain prevention intervention and can inform future weight gain prevention intervention messages. In addition, the classification of bouts had a marked effect on the prevalence estimates of those meeting physical activity recommendations. More research is needed to understand how to present both physical activity and dietary changes in weight gain prevention interventions and how to stress the importance of physical activity for overall health.

## Abbreviations

ANCOVA: Analysis of covariance
BMI: Body mass index
FPL: Federal poverty level
MVPA: Moderate-vigorous physical activity
PA: Physical activity
